# Exploring the interface zone in breast cancer: implications for surgical strategies and beyond

**DOI:** 10.1186/s13058-023-01734-0

**Published:** 2023-11-03

**Authors:** Kefah Mokbel, Munaser Alamoodi

**Affiliations:** 1 Princess Grace Hospital, The London Breast Institute, London, W1U 5NY UK; 2 Faculty of Medicine, King Abdulaziz University, Jeddah, Saudi Arabia

We read with interest the article by Wang et al. [1], in which the authors demonstrated tumor-promoting features in the microenvironment of the interface zone of breast cancer. Their elegant work is commendable, and we concur with their findings. However, their suggestion that removal of the interface zone (i.e., 5 mm margin width) would reduce the risk of breast cancer relapse is not supported by clinical data.

Over time, there has been a gradual de-escalation of breast cancer  surgery, moving from mastectomy to adequate local excision followed by radiation therapy. Furthermore, international guidelines now advocate for a tumor-free margin of 1mm. It is plausible that adjuvant radiation and systemic therapy can modify the interface zone, making complete resection of the zone less beneficial [2]. Moreover, recent evidence has shown that breast-conserving therapy is associated with superior overall survival compared to mastectomy [2]. The latter surgical approach removes the interface zone in its entirety. We believe this observation is related to the emerging understanding that breast cancer is a systemic disease, even in its early stages, as supported by the detection of circulating tumor cells [3]. These cells, when reactivated, can infiltrate the index breast quadrant and cause local recurrence near the interface zone. This path is more accessible for these cells than establishing a new premetastatic niche in distant organs, which is a more serious clinical event. It is likely that the CXCR4/SDF-1 axis mediates the chemotaxis of the circulating tumor cells toward the interface zone, thus explaining why 95% of local recurrences occur in the index quadrant [4]. Our hypothesis suggests that total mastectomy would be associated with a higher risk of distant metastasis and mortality [2], and a lower risk of locoregional recurrence [5] compared with breast-conserving surgery, and this aligns with clinical observations [2, 5].

## Data Availability

Not applicable.
